# Exploring countermovement jump variables across competitive levels and playing positions in futsal

**DOI:** 10.3389/fspor.2024.1371467

**Published:** 2024-02-23

**Authors:** Konstantinos Spyrou, Pedro E. Alcaraz, Antonio Martínez-Serrano, Elena Marín-Cascales, Davide Ferioli, Jean Paul Santis Contreras, João Nuno Ribeiro, Bruno Travassos, Tomás T. Freitas

**Affiliations:** ^1^ UCAM Research Center for High Performance Sport, UCAM Universidad Católica de Murcia, Murcia, Spain; ^2^ Facultad de Deporte, UCAM Universidad Católica de Murcia, Murcia, Spain; ^3^ Strength and Conditioning Society, Murcia, Spain; ^4^ Research Center in Sports Science, Health Sciences and Human Development (CIDESD), Department of Sport Sciences, University of Beira Interior, Covilhã, Portugal; ^5^ Portugal Football School, Portuguese Football Federation, Lisbon, Portugal; ^6^ NAR Nucleus of High Performance in Sport, São Paulo, Brazil

**Keywords:** five-a-side soccer, team-sports, professional, playing role, vertical jump

## Abstract

**Introduction:**

The aims of this study were to compare several countermovement jump (CMJ) kinetic variables between professional (PRO) and semi-professional (SEMI-PRO) futsal players and examine the differences amongst playing positions.

**Methods:**

CMJ performance from 56 male futsal players (25.2 ± 4.8 years; weight: 74.4 ± 6.4 kg) was analysed. Players were separated into PRO (*n* = 29; 27.0 ± 4.4 years; 75.4 ± 6.0 kg) and SEMI-PRO (*n* = 27; 22.7 ± 4.3 years; 73.1 ± 6.8 kg), and according to playing position: defenders (*n* = 16; 25.4 ± 3.7 years; 75.2 ± 6.0 kg), wingers (*n* = 26; 23.5 ± 4.5 years; 72.0 ± 6.9 kg), and pivots (*n* = 14; 28.0 ± 5.6 years; 77.8 ± 4.3 kg). Linear mixed models and effect sizes were used for the analyses based on the mean of two jumps for each variable.

**Results:**

PRO players presented a deeper center of mass (COM) displacement (*p* = 0.002, ES = 0.83), greater eccentric (Ecc) absolute (*p* = 0.019, ES = 0.61) and relative peak power (*p* = 0.046, ES = 0.52), and achieved greater Ecc peak velocities (*p* = 0.004, ES = 0.76) when compared to SEMI-PRO. Non-significant and trivial-to-small differences were observed in all the other CMJ variables according to the competitive level and playing position.

**Discussion:**

Ecc capabilities (i.e., deeper COM displacement, greater Ecc absolute and relative peak power, and peak velocity) during vertical jump seem to differentiate PRO and SEMI-PRO players. However, CMJ variables do not discriminate amongst playing positions in futsal players.

## Introduction

Futsal, also known as five-a-side indoor soccer, is a team-sport officially authorized by Fédération Internationale de Football Association (FIFA) that is becoming increasingly popular as evidenced by the great number of futsal-related research in the last years. Despite its apparent similarities with football (i.e., soccer), futsal can be distinguished in numerous aspects such as the playing field dimensions (40 × 20 m rubber or parquet court vs. 110 × 60 m artificial or natural grass), number of players, unlimited substitutions, and game time. Futsal is considered as a high-intensity intermittent team-sport in which professional (PRO) players cover a total distance of ∼3,750 m, of which ∼675 m are spent running (12–18 km·h^−1^) and ∼135 m sprinting (>18 km·h^−1^) and perform a great number of accelerations, decelerations, changes of direction, and explosive movements (1–3). Moreover, compared to semi-professional (SEMI-PRO) players (e.g., national state team), PRO athletes (e.g., international level team) cover a 42% greater total distance (∼4,300 m vs. ∼3,000 m), and complete a higher number of sideways or backward movements, and total overall activities (i.e., ∼470 vs. ∼310) (4). For this reason, well-developed physical capabilities play a crucial role in futsal, as they allow players to cope and reach higher match demands (2).

Regarding neuromuscular performance, several researchers (5–10) found that futsal PRO players significantly outperform SEMI-PRO in sprint, repeated sprint ability, standing broad jump, and change of direction and reactive agility tests. This phenomenon could contribute, at least in part, to the differences in overall match performance and injuries between PRO and SEMI-PRO futsal players. Remarkably, when it comes to jumping ability, players from different competition levels have been reported to present similar countermovement jump (CMJ) height values (7, 9, 11). However, CMJ height alone may not be sensitive enough to analyse an athlete's neuromuscular characteristics (i.e., explosiveness, fatigue, adaptation, etc.) or to detect changes in jump strategy or deviations in technique (eccentric [Ecc]—concentric [Con] phase metrics) as opposed to other CMJ metrics (12, 13). For instance, the analysis of specific Ecc variables may provide valuable information regarding the presence of neuromuscular fatigue or potential adaptations induced by acute/chronic training or competition stimuli (12, 13). Furthermore, different CMJ metrics may offer complementary insights since peak force and power have been suggested to be strongly associated with strength, linear speed and change of direction ability, while time-based metrics [e.g., modified reactive strength index (RSImod)] appear to be more sensitive to neuromuscular fatigue (12, 14). These observations support the notion that a more thorough analysis of the CMJ may help strength and conditioning coaches prescribe proper tailor-made training or recovery plans adapted to players’ performance, fatigue and return-to-play from injury status (12). Moreover, such analysis of the kinetic variables during the jump-land cycle in both PRO and SEMI-PRO futsal players is warranted, particularly considering that: (1) futsal specific actions are strongly associated with high-intensity accelerations-decelerations (15) that express Con and Ecc capabilities, respectively; and (2) vertical force production plays a crucial role in athletic actions, such as sprinting and change of direction (14).

Considering players' positional demands, recent studies (16–18) demonstrated that match activities vary amongst positions (i.e., defenders, wingers, and pivots). However, in the individual analysis of players capacities Caetano et al. (19) found no match demands positional differences in terms of sprint distance, peak velocity, recovery time between consecutive sprints, and number of sprints per minute. Similarly, the evaluation of jumping ability (i.e., CMJ height) amongst playing positions in futsal (10), reported non-significant differences between positions. Once again, no additional CMJ metrics were analyzed, suggesting that futsal practitioners could benefit from a more thorough playing position-specific analysis of the neuromuscular performance, to further understand its specificity in terms of force production.

To date, no studies have analyzed the differences in CMJ kinetic variables according to competition level (i.e., PRO vs. SEMI-PRO) and playing position (i.e., defenders, wingers and pivots). This information may be important to fill a gap in the literature, and help futsal practitioners optimize training practices, long-term player physical development, and talent identification by potentially highlighting neuromuscular characteristics that discriminate players from higher competition levels or with a specific positional role. Therefore, the aims of this study were to compare several CMJ metrics [i.e., CMJ height, center of mass (COM) displacement, RSImod, and Ecc and Con duration, peak force, power, and velocity] between PRO and SEMI-PRO futsal players, and to analyze the differences in the above-mentioned metrics among playing positions (i.e., defenders, wingers and pivots). According to the futsal match demands highlighted in the literature, it was hypothesized that: (1) PRO players would present higher performance in all CMJ metrics when compared to SEMI-PRO players; and (2) no differences would be observed between playing positions due to the tactical and technical characteristics of the sport that require players from different positions to engage in similar game actions (20).

## Methods and materials

### Study design

A retrospective study was designed address the research question focused on comparing the CMJ kinetics metrics between PRO and SEMI-PRO futsal players and amongst playing positions. All players were evaluated once after the pre-season period (i.e., September) during the seasons 2019–2020 and 2021–2022. This period was selected to ensure that all the players were tested in the same phase of the season (i.e., right after the pre-season and before the beginning of the in-season period) in order to avoid the influence of individual playing time and different load distribution across the competitive period. To be included in the study all players had to be: (1) on-court players (i.e., goalkeepers were not included in this study); (2) evaluated in the same period under the instruction of the same researcher and using the same force platform; (3) free from injury in the previous three months and; (4) complete the standard training program of their respective team during the weeks preceding the test session. CMJ data were collected following a standardized general warm-up protocol consisting of running-based activities (i.e., 5 min treadmill running), dynamic stretching, and core and lower-body activation exercises (2 sets × 12 repetitions of bodyweight squat and lunges), followed by a test-specific warm-up (i.e., 2 repetitions of sub-maximal CMJ attempts). All evaluations were completed at the same time of the day, in the same facilities and following at least 24 h of rest (i.e., training day-off) to avoid any acute or residual fatigue effects.

### Participants

Fifty-six male futsal players (age: 25.2 ± 4.8 years; body mass: 74.4 ± 6.4 kg) were recruited from 4 different teams and classified as PRO or SEMI-PRO according to their competitive level. The former group consisted of 29 players (age: 27.0 ± 4.4 years; body mass: 75.4 ± 6.0 kg) that competed in the 1st Division of Spain [Liga Nacional de Fútbol Sala (LNFS)] whereas the latter consisted of 27 players (age: 22.7 ± 4.3 years; body mass: 73.1 ± 6.8 kg) competing in either the 2nd Division of Spain (*n* = 8), or the 2nd B Division of Spain (*n* = 19). Furthermore, all players were separated per position as follows: 16 defenders (age: 25.4 ± 3.7 years; body mass: 75.2 ± 6.0 kg), 26 wingers (age: 23.5 ± 4.5 years; body mass: 72.0 ± 6.9 kg), and 14 pivots (age: 28.0 ± 5.6 years; body mass: 77.8 ± 4.3 kg). All players provided individual consent for data collection and study participation. All procedures were approved by the Local Ethics Committee with the registration number CE072008 and conducted according to the Declaration of Helsinki.

### Procedures

Vertical Jump Test: Players performed the CMJ test on a portable force platform (Kistler 9286BA, Kistler Group, Winterthur, Switzerland). All data were exported and analysed with a specific software (ForceDecks, Vald Performance, Brisbane, Australia). Players were required to perform a downward movement followed by a complete, rapid extension of the lower-limbs. The depth of the countermovement was self-selected to avoid changes in jumping coordination, with the aim of ensuring greater ecological validity. The hands were placed on the hips throughout the whole movement and athletes were directed to jump as high as possible and land close to the take-off point. They executed two maximal trials with 1 min rest and the mean of the two jumps was retained for analysis. The following variables were selected: CMJ height, COM displacement, RSImod, braking duration-contraction time, deceleration duration, and Ecc and Con duration, peak force, power, and velocity, in line with the previous study (12, 13) recommendations to monitor players' performance profile (Table 1). A total of 64 individual CMJ samples were analyzed, as some participants were assessed both seasons.

**Table 1:** Determination of each phase and metric during the countermovement jump.

Phases	Description
Eccentric	Negative velocity starting from point where a 20 N threshold is exceeded until velocity = 0 m·s^−1^
Braking	Eccentric subphase: point of minimum force until velocity = 0 m·s^−1^
Deceleration	Eccentric subphase: peak negative velocity to 0 m·s^−1^
Concentric	Positive velocity from = m·s^−1^ until takeoff
Flight	From when vGRF falls below 30 N until vGRF return to above 30 N
Variables	Description
Jump height (cm)	Maximal jump height computed using impulse-momentum method
RSImod (m/sec)	Jump height (calculated from flight time) divided by contraction time
Braking duration—Contraction Time	Duration of the braking phase divided by contraction time
COM Displacement (cm)	Maximal vertical center of mass displacement during initial ground contact
Deceleration duration (ms)	Time period from maximum negative velocity to zero velocity at the end of the eccentric phase
Eccentric Duration (ms)	Duration of the eccentric phase
Eccentric Peak Force ABS (N)	Greatest force achieved during the eccentric phase
Eccentric Peak Force REL (N)	Greatest force achieved during the eccentric phase relative to the athletés weight
Eccentric Peak Power ABS (W)	Greatest power achieved during the eccentric phase
Eccentric Peak Power REL (W)	Greatest power achieved during the eccentric phase relative to the athletés weight
Eccentric Peak Velocity (m/s)	Greatest velocity achieved during the eccentric phase
Concentric Duration (ms)	Duration of the concentric phase
Concentric Peak Force ABS (N)	Greatest force achieved during the concentric phase
Concentric Peak Force REL (N)	Greatest force achieved during the concentric phase relative to the athletés weight
Concentric Peak Power ABS (W)	Greatest power achieved during the concentric phase
Concentric Peak Power REL (W)	Greatest power achieved during the concentric phase relative to the athletés weight
Concentric Peak Velocity (m/s)	Greatest velocity achieved during the concentric phase

ABS, absolute; COM, center of mass; Dec, deceleration; REL, relative; RSI_mod_, reactive strength index modified; vGRF, vertical ground reaction force.

### Statistical analysis

The results are reported as estimated marginal means with 95% confidence intervals. Before running linear mixed models, boxplots and histograms were used to identify and exclude potentially influential data points using the interquartile method. No outliers were detected in the analysis. Following this procedure, residual plots were visually inspected to determine deviations from homoscedasticity or normality. All assumptions were satisfied (i.e., homoscedasticity and normality *p* value > 0.05), and the normality of the residuals was also assessed using the Kolmogorov-Smirnov test. Subsequently, linear mixed models were constructed to examine differences in CMJ variables according to competitive level and playing position, accounting for individual repeated measures. In all linear mixed models, competitive level (two levels) and playing position (three levels) were used as fixed effect and player as random effect with a random intercept and fixed slope. All assumptions were met, and the normality of the residuals was assessed using the Kolmogorov-Smirnov test. Pairwise comparisons were performed using the Bonferroni post-hoc analysis. The t statistics from the mixed model were converted into Cohen's d effect sizes and associated 95% confidence intervals. Effect sizes were interpreted as follows: <0.2, trivial; 0.20–0.59, small; 0.60–1.19, moderate; 1.2–1.99, large; and ≥2.0, very large (21). An alpha level of *p* ≤ 0.05 was set *a priori* for statistical significance. All tests used in this study displayed high levels of absolute and relative reliability (i.e., intraclass correlation coefficients >0.90 and coefficients of variation <10%). All data were analyzed using a statistical package (Jamovi, version 1.8, 2,021).

## Results

Descriptive data and statistical analyses for CMJ kinetic variables according to competitive level are presented in Table 2. PRO players displayed greater COM displacement (*p* = 0.002, ES = 0.83, moderate), higher Ecc absolute (*p* = 0.019, ES = 0.61, moderate) and relative peak power (*p* = 0.046, ES = 0.52, small), and greater Ecc peak velocities (*p* = 0.004, ES = 0.76, moderate) when compared to SEMI-PRO. Non-significant and trivial-to-small differences were observed in all other CMJ variables (Ecc and Con phase) according to the competitive level.

**Table 2:** Comparison of countermovement jump variables according to competitive level.

Dependent variable (units)	EMMeans (95% CI)		ES (95% CI)	Interpretation	*p* value
	PRO	SEMI-PRO			
Jump height (cm)	36.6 (35.1; 38.1)	35.9 (34.3; 37.5)	0.16 (−0.33; 0.66)	Trivial	0.516
RSI_mod_ (m/sec)	0.514 (0.483; 0.546)	0.506 (0.473; 0.540)	0.09 (−0.41; 0.58)	Trivial	0.726
Eccentric (“downward”) phase					
Braking duration -Contraction Time	40.4 (38.6; 42.2)	41.1 (39.1; 43.1)	0.14 (−0.35; 0.64)	Trivial	0.577
**COM Displacement (cm)**	**32.7** **(34.3; 31.2)**	**28.9** **(30.6; 27.3)**	**0.83** **(0.31; 1.34)**	**Moderate**	**0** **.** **002** *
Dec duration (ms)	173 (161; 185)	155 (142; 168)	0.51 (0.01; 1.01)	Small	0.050
Duration (ms)	487 (461; 514)	476 (447; 504)	0.15 (−0.34; 0.65)	Trivial	0.544
Peak Force ABS (N)	1,790 (1,702; 1,877)	1,771 (1,678; 1,864)	0.07 (−0.42; 0.57)	Trivial	0.774
Peak Force REL (N)	23.6 (22.7; 24.5)	24.0 (23.1; 25.0)	0.15 (−0.35; 0.64)	Trivial	0.560
** Peak Power ABS (W)**	**1,449** **(1,315; 1,584)**	**1,211** **(1,067; 1,356)**	**0.61** **(0.10; 1.11)**	**Moderate**	0.019*
** Peak Power REL (W)**	**19.1** **(17.4; 20.9)**	**16.5** **(14.6; 18.4)**	**0.52** **(0.01; 1.02)**	**Small**	0.046*
** Peak Velocity (m/s)**	**1.32** **(1.38; 1.25)**	**1.18** **(1.25; 1.11)**	**0.76** **(0.25; 1.27)**	**Moderate**	0.004*
Concentric (“upward”) phase					
Duration (ms)	262 (251; 273)	249 (237; 261)	0.41 (−0.03; 0.91)	Small	0.107
Peak Force ABS (N)	1,857 (1,777; 1,937)	1,836 (1,751; 1,921)	0.09 (−0.40; 0.59)	Trivial	0.719
Peak Force REL (N)	24.5 (23.8; 25.3)	24.9 (24.1; 25.8)	0.18 (−0.31; 0.68)	Trivial	0.475
Peak Power ABS (W)	4,041 (3,866; 4,216)	3,930 (3,745; 4,114)	0.22 (−0.27; 0.72)	Small	0.384
Peak Power REL (W)	53.3 (51.5; 55.2)	53.5 (51.6; 55.4)	0.03 (−0.46; 0.53)	Trivial	0.898
Peak Velocity (m/s)	2.79 (2.74; 2.84)	2.77 (2.71; 2.82)	0.13 (−0.36; 0.62)	Trivial	0.609

ABS, absolute; CI, Confidence Interval; COM, center of mass; Dec, deceleration; ES, effect size; EMMeans, estimated marginal means; REL, relative; RSImod, reactive strength index modified.

*Bolded *p* value indicates statistically significant difference (*p* < 0.05).

Descriptive data and statistical analyses for CMJ kinetic metrics according to playing position are presented in Table 3. No statistically significant differences (*p* > 0.05, ES ranging from 0.00 to 0.51, trivial-to-small) were observed in any of the CMJ variables when comparing among positions.

**Table 3:** Comparison of countermovement jump variables according to playing position.

Dependent variable (units)	EMMeans (95% CI)			Main effect *p* value	W vs. D		W vs. P		D vs. P	
	Winger	Defenders	Pivot		ES (95% CI)	*p* value	ES (95% CI)	*p* value	ES (95% CI)	*p* value
Jump height (cm)	37.3 (35.8; 38.9)	35.1 (33.1; 37.1)	36.3 (34.2; 38.5)	0.217	0.51 (−0.06; 1.09)	0.083	0.24 (−0.39; 0.87)	0.464	0.29 (−0.37; 0.96)	0.391
RSI_mod_ (m/sec)	0.532 (0.500; 0.565)	0.499 (0.457; 0.541)	0.499 (0.455; 0.544)	0.341	0.36 (−0.21; 0.93)	0.214	0.38 (−0.25; 1.01)	0.239	0.00 (−0.67; 0.66)	0.989
Eccentric (“downward”) phase										
Braking duration—Contraction Time	41.3 (39.4; 43.2)	41.7 (39.3; 44.0)	39.3 (36.7; 41.9)	0.359	0.07 (−0.49; 0.64)	0.801	0.39 (−0.24; 1.03)	0.225	0.45 (−0.22; 1.12)	0.187
COM Displacement (cm)	30.9 (32.6; 29.3)	30.2 (32.3; 28.1)	31.3 (33.6; 29.1)	0.742	0.16 (−0.41; 0.73)	0.581	0.09 (−0.53; 0.72)	0.768	0.25 (−0.41; 0.92)	0.456
Dec duration (ms)	164 (152; 177)	161 (145; 177)	166 (149; 184)	0.892	0.11 (−0.46; 0.67)	0.717	0.05 (−0.58; 0.68)	0.876	0.15 (−0.51; 0.82)	0.649
Duration (ms)	478 (450; 506)	467 (432; 502)	500 (462; 538)	0.430	0.14 (−0.42; 0.71)	0.618	0.30 (−0.33; 0.93)	0.349	0.44 (−0.23; 1.11)	0.203
Peak Force ABS (N)	1,741 (1,650; 1,831)	1,766 (1,650; 1,881)	1,835 (1,711; 1,958)	0.470	0.10 (−0.47; 0.67)	0.732	0.39 (−0.24; 1.03)	0.223	0.28 (−0.39; 0.94)	0.417
Peak Force REL (N)	24.2 (23.3; 25.2)	23.7 (22.6; 24.9)	23.5 (22.3; 24.8)	0.636	0.19 (−0.38; 0.76)	0.516	0.28 (−0.35; 0.91)	0.379	0.08 (−0.58; 0.74)	0.812
Peak Power ABS (W)	1,288 (1,148; 1,429)	1,374 (1,196; 1,551)	1,329 (1,137; 1,521)	0.750	0.22 (−0.35; 0.79)	0.453	0.11 (−0.52; 0.74)	0.733	0.12 (−0.55; 0.78)	0.733
Peak Power REL (W)	18.1 (16.2; 19.9)	18.4 (16.1; 20.7)	17.0 (14.5; 19.5)	0.694	0.07 (−0.50; 0.64)	0.811	0.22 (−0.41; 0.85)	0.502	0.28 (−0.39; 0.94)	0.414
Peak Velocity (m/s)	1.26 (1.32; 1.19)	1.25 (1.34; 1.17)	1.23 (1.32; 1.14)	0.879	0.02 (−0.55; 0.58)	0.950	0.16 (−0.47; 0.79)	0.624	0.13 (−0.53; 0.80)	0.697
Concentric (“upward”) phase										
Duration (ms)	253 (241; 264)	252 (237; 266)	263 (247; 279)	0.513	0.02 (−0.54; 0.59)	0.935	0.33 (−0.30; 0.97)	0.300	0.35 (−0.32; 1.01)	0.311
Peak Force ABS (N)	1,800 (1,717; 1,883)	1,834 (1,729; 1,940)	1,904 (1,791; 2,017)	0.334	0.15 (−0.42; 0.72)	0.607	0.48 (−0.16; 1.11)	0.141	0.31 (−0.36; 0.97)	0.368
Peak Force REL (N)	25.1 (24.3; 25.9)	24.7 (23.7; 25.7)	24.4 (23.3; 25.5)	0.611	0.18 (−0.39; 0.74)	0.540	0.31 (−0.33; 0.94)	0.344	0.11 (−0.55; 0.78)	0.738
Peak Power ABS (W)	3,928 (3,748; 4,108)	3,929 (3,698; 4,159)	4,099 (3,854; 4,345)	0.485	0.00 (−0.57; 0.57)	0.999	0.36 (−0.27; 0.99)	0.264	0.34 (−0.32; 1.01)	0.313
Peak Power REL (W)	54.8 (52.9; 56.7)	53.0 (50.6; 55.3)	52.6 (50.0; 55.1)	0.294	0.35 (−0.22; 0.92)	0.233	0.45 (−0.19; 1.08)	0.169	0.07 (−0.59; 0.74)	0.828
Peak Velocity (N)	2.81 (2.76; 2.87)	2.74 (2.67; 2.81)	2.78 (2.71; 2.85)	0.257	0.48 (−0.09; 1.05)	0.102	0.23 (−0.39; 0.86)	0.466	0.26 (−0.40; 0.93)	0.441

BS, absolute; COM displacement, centre of mass displacement; Dec, deceleration: D, defense; EMMeans, estimated marginal means; REL, relative; RSImod, reactive strength index modified; P, pivot; W, winger.

## Discussion

The aim of this study was to compare several CMJ metrics between PRO and SEMI-PRO futsal players and analyze the differences in the above-mentioned metrics among playing positions. The main findings were that: (1) PRO players displayed superior Ecc capabilities, performing a higher COM displacement, generating greater absolute and relative peak power, and achieving greater peak velocities during the Ecc phase when compared to SEMI-PRO players. As expected, non-significant differences were found in any of the CMJ variables when considering playing positions.

Regarding jumping ability comparison between competition levels, previous studies (7, 9) found that PRO players presented similar CMJ height values when compared to SEMI-PRO players, aligning with the findings obtained in this study. This implies that CMJ height alone may not be the most suitable metric to discriminate players' profiles, the competitive level or to be used for talent identification purposes. Conversely, when conducting a more comprehensive analysis of the kinetic variables during the jump-land cycle, PRO players displayed superior outcomes in several metrics of the Ecc (i.e., downward) phase (i.e., COM displacement, Ecc absolute and relative peak power, and Ecc peak velocity) than their lower-level counterparts. The observed differences according to the competitive level indicate that PRO players have a better ability to produce higher levels of forces on shorter time frames. Accordingly, despite no differences were observed in peak forces (both absolute and relative) between PRO and SEMI-PRO players, the former group was characterized by greater levels of power (both absolute and relative) and greater peak velocities during the Ecc phase. These capacities may play a key role during the futsal specific movements and contribute to be more efficient during the match-play from a physical point of view. The superior level of preparation but also the higher number of matches and training sessions that PRO players are exposed to (approximately 50 vs. 30 games per competitive season and around 6 vs. 3 training sessions per week) when compared to SEMI-PRO could, at least in part, explain the differences observed. It is important to highlight that PRO players must cope with higher physical match-demands and had more years of experience performing specific movement patterns of the sport, thus, potentially developing superior Ecc capabilities, stretch-shortening cycle mechanisms and muscle-tendon properties compared to lower-level players (22, 23). From an applied perspective, the present results suggest that: (1) futsal players may benefit from performing Ecc-based and plyometric exercises (e.g., flywheel training or drop jumps, respectively), thus producing high levels of force within short time periods during resistance training sessions; and (2) a more comprehensive analysis of CMJ is recommended to evaluate and compare players from different competitive levels.

When comparing vertical jump ability amongst playing positions, non-significant trivial-to-small differences were found in all CMJ metrics. Results support previous results (10), which suggests that vertical jump seems not to differentiate futsal players from different positions, and expand current knowledge by reporting no differences in a multitude of complementary jump-land variables. To some extent, the similar performances observed in all CMJ metrics among on-court players could be explained by the fact that, in futsal, playing positions are not as clearly define as in other indoor sports [e.g., basketball (24) or handball (25)]. In fact, in futsal, tactical behaviors usually require players to adopt multiple playing positions (20) and a multitude of individual tactical actions that are essentially characterized by mechanical demands (high acceleration and deceleration). That is, futsal actions, independently of the players' positions, are strongly associated with similar high intensity physical demands, despite the different tactical role of playing positions (15). Thus, an individualized perspective to assess players' profiles is required in order to better sustain their capacities according to their profile of play and this phenomenon could help to review the current training models, encouraging for a more individualized positioning approach to physical conditioning in futsal. Future studies should further investigate the relationship between the most key determinants factors of performance (e.g., technical-tactical, physical, and anthropometrical characteristics) for player’s position in futsal.

This study is limited by the fact that CMJ data were collected only at the end of the pre-season period (i.e., September), which does not allow us to conclude whether similar results would be obtained during the most crucial moments of the season (i.e., competitive period); nevertheless, we decided to utilize this time-point in order to avoid the effects of individual playing time and different load distribution during the competitive period. Thus, additional studies in futsal analyzing the neuromuscular performance across the season and how it fluctuates in both PRO and SEMI-PRO players is necessary. Moreover, when dividing the sample into playing position, a small sample size was analyzed in each group, which may have precluded us from identifying clear between-group differences. Consequently, more research with a higher sample size, and collaborations from different leagues and countries is warranted to have a clear view concerning the differences between futsal playing positions. Lastly, all the subjects competed in Spain and the results of the extrapolation of the present findings to other populations should be done with caution. However, this data could be used as an initial benchmark for other researchers to differentiate between PRO and SEMI-PRO futsal players. Future research should incorporate more physical assessments, such as sprint, change of direction, isometric mid-tight pull, and strength deficit calculations to better characterize PRO and SEMI-PRO players' neuromuscular performances.

In conclusion, the use of CMJ to evaluate futsal players capacities should consider not only jump height but also different Ecc and Con kinetic variables. The Ecc capacity seems to discriminate PRO from SEMI-PRO players, as seen by the deeper COM displacement, the greater absolute and relative Ecc peak power, and the highest Ecc peak velocity of the former. In practical terms, Ecc capacity plays a crucial role in futsal and may help to distinguish between players from different levels of competition. Thus, it should be included in both futsal evaluation batteries and physical development programs. Practitioners should be aware of the importance of enhancing players' Ecc capacity via futsal specific drills, such as small sided games with a great number of accelerations-decelerations or Ecc-based resistance and plyometric training. Lastly, CMJ seems to not be capable to discriminate playing positions in futsal due to the similarity in game requirements for the different positions. However, these results could help to review the current training models, encouraging for a more individualized approach to physical conditioning in futsal.

## Data Availability

The raw data supporting the conclusions of this article will be made available by the authors, without undue reservation.
